# Establishing a framework to understand the regulation and control of dogs in urban environments: a case study of Melbourne, Australia

**DOI:** 10.1186/s40064-016-2843-8

**Published:** 2016-07-27

**Authors:** Simon Bruce Carter

**Affiliations:** Faculty of Architecture, Building and Planning, University of Melbourne, Melbourne, Victoria Australia

**Keywords:** Dogs, Animal management, Regulation, Local government, Institutional ontology

## Abstract

**Background:**

This study examines the effectiveness of animal management from a critical theory perspective, establishing a framework to describe the animal management activities of local government. In creating sustainable cities, local government must critically engage with the management of other species which live alongside humans. Despite around 40 % of Australian households owning a dog, there is relatively scarce scholarly attention paid to animal management as a subject in its own right. There are numerous studies examining the need to regulate dogs, however there are relatively few studies which examine the effectiveness of regulation.

**Results:**

This study adopts interpretive qualitative content analyses of documentary and interview accounts to critically describe the practice of animal management and suggest why it takes place the way it does. An ontological-methodological framework is introduced to frame the practice of animal management, relating the methodology of animal management to the underlying ontological orientation of local government. This study highlights some institutional conditions which allow particular animal management activities to flourish. Enforcement of barking dog nuisance and responsible dog ownership education are shown to demonstrate attributes of regulatory success. Conversely, enforcement of effective control and community education processes demonstrate some attributes of regulatory failure.

**Conclusions:**

This study demonstrates how institutional ontology and methodology affect the practice of animal management. This study provides animal management officers and local government with a means to critically examine particular approaches to animal management in practice, offering an opportunity to improve the effectiveness of animal management functions in local government. In contributing to improving the awareness of local government as to how they plan for and manage dogs, this study contributes to a broader community consideration of dogs as a beneficial part of society.

## Background

Urban environments are a principally human habitat built by humans to satisfy human objectives (Tarsitano 2006). Cities can be thought to exist as part of a broader ecosystem (Bender 2010), a ‘natural’ environment where a multitude of plants and animals live and perhaps flourish alongside humans (Sanders 2011). The process of domestication has brought dogs to dwell alongside humans to live in our communities and, by extension, share our habitat (Howell 2012). Their needs are subsumed by the needs of the broader community as their use of the public realm becomes increasingly contested (Lee et al. 2009); however the tide is shifting as the needs of dogs are becoming more recognised by urban dwellers (Urbanik and Morgan 2013). These needs are not always aligned, leading to contest and perhaps conflict. Regulations exist to manage this interaction, on the one hand regulating how dogs and their owners actually behave and on the other reflective of and reflexive toward community expectations of how dogs and their owners ought to behave. This paper examines the integration of the dog in the urban environment through the narrow lens of these regulations and controls, in turn seeking to understand why certain approaches to the regulation and control of dogs appear more effective than others in practice.

In his essay examining research in comparative politics, Hall (2003) postulates that ontology and methodology are co-dependent. This paper describes animal management as similarly dichotomous, comprising respectively an ontological function (process and outcomes) and a methodological function (education and enforcement). The ontological function describes the nature of an underlying motivation for animal management whereas the methodological function describes how animal management is practiced. This provides a valuable framework to critically appraise the management and regulation of dogs in order to understand why some regulation is more effective than others, yielding a number of recommendations for animal management regulation, policy and practice regarding dog management in the community.

Dog ownership is popular in Australia with around 39 % of households owning a dog (Richmond 2013). Dogs are increasingly recognised as having both a private and public life, where their needs ought to be recognised beyond the private realm and in the public realm (Urbanik and Morgan 2013). As a consequence, dogs then are increasingly mixing and in some cases conflicting with human society, necessitating a form of regulation.

In his examination of nineteenth century regulation of dogs in London, Howell (2012) at page 222 describes the ‘problem of the public dog’ and delineates government responses into two branches, disciplining treatments controlling what dogs are allowed to do in the public realm and regulating treatments describing acceptable behaviours of dogs and their owners, the latter especially expressing speciesist power. Speciesism is rife in regulation of dogs in urban areas. Even regulations which promote the welfare of dogs seek to elevate the human species to decide which freedoms a dog is allowed (Srinivasan 2013). That is, our ‘caring’ is essentially an act of subjugation and superiority. We choose to regulate behaviours of dogs in public open space more than we regulate behaviours of humans (Urbanik and Morgan 2013), these choices describing an anthropocentrism in how we admit dogs into ‘our’ urban habitat. Tardona (2012) on the other hand interprets such regulation of dogs in public open space as beneficial for dogs since it both improves canine welfare and contributes to community harmony. Government responses change over time in response to shifts in community values; in a case study from the state of New South Wales, Australia, Borthwick (2009) maps a cultural shift in treatment from the disciplinary to the regulatory over 1966–1998. This paper alludes to a similar contemporary regulatory focus (and perhaps a shift in focus) in the neighbouring state of Victoria too.

We remain a long way from Wolch’s (1996) *Zoöpolis*. Nature remains ‘the other’ and dogs are not a part of the human habitat of the city; their accommodation into human culture needs to be regulated. Instone and Sweeney (2014) examine the emergence of dog culture in Australia through a lens of the ‘dog-as-problem’, contending that the problem with dogs in public is not one of control or regulations rather a clash of agency with anthropocentric desire. As humans decide how they exercise this agency of regulation, they exercise anthropocentrism by choosing what they regulate, how they regulate and whether they later conform to those regulations (Rohlf et al. 2010); in other words, responsible dog ownership and compliance with regulations need not always coincide. Regulating dogs in public open space typically focuses on the dog and the dog owner, ignoring any reference to responsibilities of others in the community, pointing to an institutionalised subordination of dogs and dog owners compared to other (human) users of public open space (Instone and Mee 2011). Whilst the choice of what and how to regulate relies on an institutional value set, the choice to conform is laden with individual values and brings to the fore rationalisations as to which conflicting activities have the greatest priority to that individual (Williams et al. 2009). These decisions are quintessential ‘moral dilemmas’ and the choice to conform depends on the ‘affective proximity’ of the individual to the consequences of their decision-making (Tassy et al. 2013). Regulating dogs in urban environments is clearly a complex task.

There remains very little research into how the ‘public dog’ is regulated and any nuisance managed. Barking dogs generate a range of concerns in the private home however that barking becomes a nuisance when it affects the public; that is, the incidence of nuisance transforms the ‘private dog’ into the ‘public dog’. A number of scientific and critical studies have been undertaken to understand the source and nature of why dogs bark (Buckland et al. 2014; Cross et al. 2009; Pongrácz et al. 2010; Yin and McCowan 2004). There have also been a number of studies to examine how barking might be monitored and addressed, hinting at a need to critically examine regulatory treatments of nuisance barking (Bragdon and Miller 1978; Flint et al. 2013; Raglus et al. 2015). Dog bark nuisance has been examined as it affects the broader fields of housing and common law (Huss 2005), however the regulation of barking dog nuisance has not been researched by scholars in any comprehensive way (Flint et al. 2014).

Similar to barking dog nuisance, there exists little contemporary research into how the ‘public dog’ is regulated in public open space (Brown 2014). In terms of dogs interacting physically with the public realm, a number of studies focus on the effect which dogs have on the natural environment and suggest regulation within the narrow confines of that context. The studies of Miller et al. (2001), Lenth et al. (2008) and Schlacher et al. (2015) each emphasise a need to consider ecological values when designing regulations for dogs. Their conclusions underscore the importance of anthropocentrism to conserving ecology of Owens and Cowell (2011, ch.3). In the context of regulating dogs in public open space, Weston et al. (2014) identify a broader absence of studies which explore the effectiveness of regulations in terms of content and structure, the extent of compliance, transgression and enforcement of those regulations and how human values influence those regulations. Whereas there is a clear need for further studies, an existing body of research does exist. Flint et al. (2014) describe a strong nexus between regulation and community education. Miller and Howell (2008) on the other hand curiously delineate ‘management’ from ‘enforcement’ and opine that a softer approach may be more effective in treating nuisance. In a spatial study, Soto and Palomares (2015) find that domesticated dogs rarely stray far from boundaries adjacent to human settlements and point towards a more effective future regulatory policy direction. Williams et al. (2009) explore barriers to compliance of dog owners on beaches, finding that the decision to comply is essentially a personal value judgement. Similarly, Instone and Sweeney (2014) demark what it means to be a responsible dog owner, finding that ‘animaling’ the city is dominated by a performative identity which questions the human-animal distinction.

Tarsitano (2006) and Gaunet et al. (2014) each identify a need to study the emerging field of dogs in cities. Understanding both how regulation of dogs takes place and how that regulation might be improved is a step towards the philosophical *Zoöpolis* opined by Wolch (1996) and hopefully a better world for dogs living in urban environments.

## Methods

**Fig. 1:**
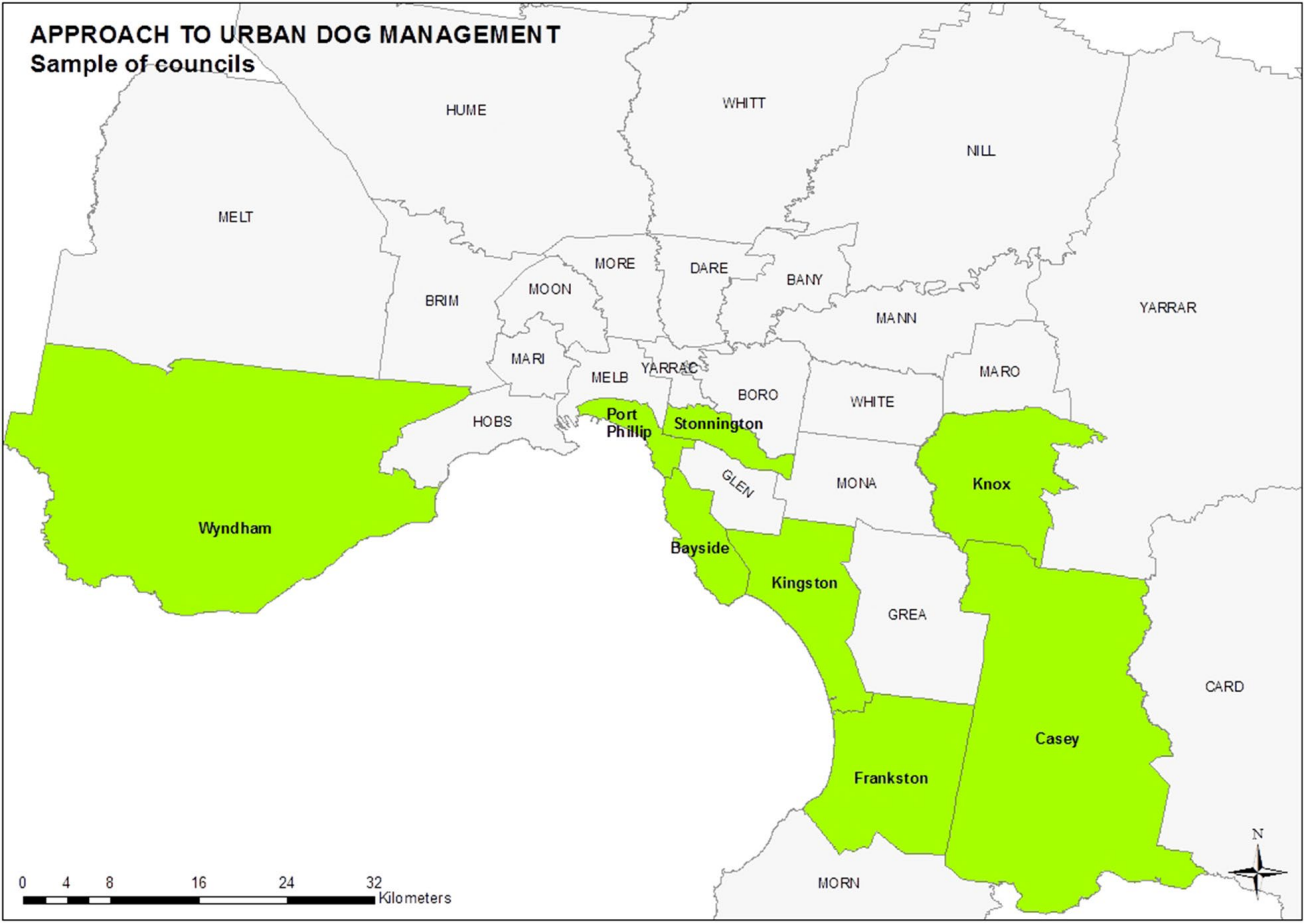
Eight representative councils from Greater Melbourne which broadly represent different approaches to animal management form the sample councils. *Source*: author, using cartographic boundary files by the Australian Bureau of Statistics (2011)

This study is based on a similar systems case study design consisting of an instrumental case of Melbourne, the capital city of the State of Victoria in Australia, with multiple internal units of analysis (Denk 2009; Denters and Mossberger 2006; Yin 2009). Australia has three distinct tiers of government—federal, state and local—each undertaking responsibilities as enshrined in the Australian Constitution and, in the case of Victoria, the *Local Government Act 1989* (Vic.). Animal management responsibilities are split between state and local government in Victoria, with the Victorian government establishing a broad legislative and policy framework and local government enacting that framework in their communities. The unit of analysis in this study is council, the object of local government authority in Victoria. Cluster sampling was used to develop the representative sample of eight councils (Krippendorff 2004). Thirty-one quantitative, qualitative and spatial characteristics of dogs living in urban environments were derived from a survey of literature. Cluster analysis was performed in two phases in order to develop the representative sample of councils (Aldenderfer and Blashfield 1984). Firstly, the characteristics underwent a reductive cluster analysis to draw out analytic meaning, this phase forming the nine thematic clusters shown in Table 1. Secondly, a selective cluster analysis determined sets of candidate councils that each fell into cross-sections of these thematic clusters. Eight councils were selected from these candidate sets as representative of Melbourne’s planning and management of dogs; these councils are depicted in Fig. 1.

**Table 1:** Thematic clusters of characteristics of councils by animal management approach derived from raw data corresponding to characteristics (Source: author)

Predominance of an *enforcement* *and* *compliance* approach to animal management	
A. Suburban environment	High interaction environment High population of adults, children and dogs, but low density Detached housing with backyards High number of incidents with dogs (including dog attacks, barking complaints and impoundments)
B. Family environment	Families with young children Home owners (rather than renters) Relatively high assets (not necessarily high income)
C. Dog ownership environment	High density of dogs to humans High rate of dog ownership
D. Larger councils	Larger councils by land area Larger number of animal management officers
Predominance of an *education* *and* *compliance* approach to animal management	
E. Affluent environment	High income High proficiency in English Relatively socioeconomically advantaged
F. Dense environment	High population density to land area High density of dogs to land area
G. Smaller councils	Smaller councils by land area Smaller number of animal management officers
H. Mature environment	Less families with young children Less detached housing and fewer backyards
No predominant approach to animal management	
I. Multicultural environment	High migrant population Fewer dogs Smaller councils by land area

Institutional data was sourced from councils and the Victorian government in the form of both written documents and semi-structured interviews with officials. Given the uncertainty introduced by anthropomorphism, individual accounts which speak of dogs typically lack consensus. Institutional traces through the voices of council and government officials have opportunity to eventually become objective and critically anthropomorphic (in the sense of Karlsson (2012)) and form a reflection of society’s attentions towards dogs that is credible, consistent and comparable. The analytical framework underlying both the data collection and analysis for this study structures around five tenets arising from an operationalisation of justice for dogs in urban environments, these resolving to five underlying drivers of council action:

- responsible dog ownership and education;
- conflict management and compliance;
- community tolerance and enforcement;
- open space planning; and
- support of the human-animal bond.

To understand the regulation and control of dogs in Melbourne, *Domestic Animal Management Plans* from 30 metropolitan councils, institutional documents and records from sample councils, and interviews with 10 animal management officers distributed across both sample councils and the Victorian state government were subject to disciplined qualitative content analyses within this analytical framework.^1^^,^^2^ The grounded theory approach is pseudo inductive-deductive, particularly whilst data continues to be collected in early stages of analysis (Corbin and Strauss 2008, ch.3; Miles and Huberman 1994, ch.4). As a whole however, the analysis fundamentally resembles the inductive, interpretative art of Krippendorff (2004) and Elo and Kyngäs (2008) rather than the deductive, scientific approach of Mayring (2000) and Kohlbacher (2006). The initial deductive contributions are absorbed into the interpretive content analysis as it takes shape.

## Discussion

The *Domestic Animals Act 1994* (Vic.) (‘the Act’) prescribes that certain processes be undertaken in the event of a limited number of particularly egregious infractions such as dog attacks. Outside of these specific provisions however, the Act affords each council substantial freedoms to manage and regulate dogs as they see fit. Every four years, each council prepares a *Domestic Animal Management Plan* which comprehensively describes its strategic priorities and planned actions in relation to the management of domesticated dogs and cats, including an evaluation of its past performance and a self-assessment of its capabilities to undertake its responsibilities under the Act. The topic matter covered within each plan is largely prescribed by the Act making the documents inherently comparable between councils.

Some councils nevertheless choose to regulate more than others, with the various approaches to animal management classified along two spectra, the methodological functioning of council regulation (educative to enforcement) and the ontological functioning of council regulation (process to outcomes). These two dimensions form a framework to describe the practice of animal management.

### Establishing the methodological functions of council regulation

Conflict arises between dogs and the community from time-to-time. A substantial majority of the examined *Domestic Animal Management Plans* describe a notional and broad goal of balancing the needs of dog owners with those of the broader community, commonly using terminology such as ‘harmonious living’ or ‘achieving a balance’ to indicate as such. Discussions of harmony are usually presented in contrast with community conflict and urban space contest, particularly concerning the incidence of community nuisance brought about by dogs and their owners, such as noise and dog litter. Harmony then becomes particularly difficult to achieve in environments where the perception of dogs is already poor.

*Domestic Animal Management Plans* have a strong foundation of compliance due to their statutory role in the Act. Regulations enshrine community expectations and values, the nature of these being subjective to the community however compliance with those regulations is objective and absolute. In practice, compliance is effected by councils through a combination of education and enforcement actions which respectively describe the reasons for regulation and the consequences of infraction. These two kinds of activity—education and enforcement—form the methodological function of council regulation. This effect is captured in *Domestic Animal Management Plans* through the systematic identification of active text which describes the *doing* of animal management, these actions generally comprising educative, compliance and enforcement activities.

**Fig. 2:**
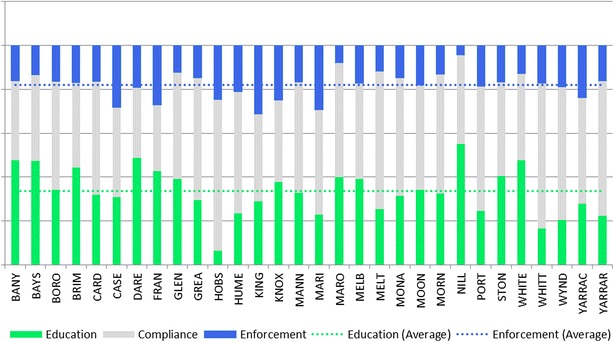
Methodological functions of 30 metropolitan councils are classified based on a qualitative content analysis of each *Domestic Animal Management Plan* (effective January 2014). The *bars* are to scale within each council and represent the relative proportion of animal management activities that are classified as ostensibly education and enforcement respectively. The respective *horizontal lines* represent the mean education and enforcement activities across all 30 councils. The *shaded* gap between the *bars* represents general compliance activities that are neither education nor enforcement focused. *Source*: author

Figure 2 shows the variability in documented approaches of each of 30 metropolitan councils (effective January 2014), painting this manifold landscape of the methodological function of animal management in practice and in turn permitting the classification of particular councils by predominant methodological function(s).

*Education activities* provide context to help the community understand what the regulation is, why it is in place and, perhaps, what happens if the regulation is contravened. Passive education performs an ‘informing’ function and does not necessarily target a particular segment of the community nor does it necessarily compel anybody to act in a particular way. Active educational approaches on the other hand perform an ‘advising’ function, these educational messages being sensitive to cultural or other local contexts and therefore typically target specific groups with relevant messages, therefore arguably able to better influence particular compliance outcomes. General *compliance activities* on the other hand prepare council to undertake its responsibilities in executing its duties under the Act. These enabling activities usually take place ‘behind the scenes’, often essential functions to allow the education and enforcement activities of council to take place. Common compliance activities include the maintenance of registration databases, training of authorised officers and activities undertaken by council officers to gain an understanding and appreciation of their community’s needs.

Whereas education activities are preventative measures to help inform or advise the community on compliance matters, *enforcement activities* are consequential measures and act on any alleged transgressions of regulations. Enforcement actions are necessarily contextualised to a particular infraction. The delineation between passive and active enforcement lies on an ordinal scale of significance of response, ranging from verbal or written warnings through to more active responses such as the issuance of infringement notices, prosecution of the owner leading to fines or incarceration and, in extreme cases, the destruction of the dog. The Act affords substantial freedom to each council in regulating animals: councils can choose to what extent which minor infractions are enforceable (what is enforced) and furthermore choose to what extent each of those infractions ought to be enforced (the nature of that enforcement). In other words, the Act allows councils the freedom to define the nature of their day-to-day animal management role. Some councils have thus adopted terminology like ‘rigid’ or ‘strict’ to establish expectations of a ‘normal’ enforcement and a ‘stronger’ enforcement in particular circumstances.

In practice, the methodological function of council regulation situates on a scale (emphasis added):

‘Community discussion and comment at these sessions reinforced the idea for increased education and awareness about responsible pet ownership, as a *complementary action* in relation to Council’s ongoing enforcement program’ (Brimbank City Council (2013) at page 34)

‘Council generally endorses a strategy of increasing awareness and understanding of the issues, developing responsible pet ownership *before* issuing penalties’ (City of Yarra (2013) at page 2)

The methodological spectrum of education to enforcement thus sketches the range of potential approaches to animal management.

### Establishing the ontological functions of council regulation

Whereas the methodological function of council regulation focuses on how regulation is enacted in practice, the ontological function of council regulation focuses on the underlying motivations for those regulatory activities in the first place. Faludi (1973) at page 5 introduces ‘levers’ and ‘effects’ of urban planning, describing a performative ontology as ranging from process through to outcomes; consequently, each council naturally identifies with either a process- or outcomes-based approach in practice.

A diverse breadth of animal management planning practices is observed across the sample councils. This is seen to largely follow the premise of Hall (2003) that ontology and methodology are co-dependent, whereby councils which a priori adopt a more enforcement-oriented approach to animal management (from their *Domestic Animal Management Plans*, depicted in Fig. 2) espouse an institutional process-oriented ontology in practice from the interview accounts of their animal management officers:

Due to our very limited resources, we generally action our issues on complaint-basis only. We have very limited proactive responses.

But I would probably argue… I think we spend most of our time working in the system, barely coping with barking dogs, dog attacks, just trying to cope with the massive amount. We’ve done a lot of work in the two years we’ve been here to actually give the officers [some] capacity… That’s working on the system, and we don’t do much of that at all, I’d be honest and say it.

Whatever the outcome is, but the outcome would suit the issue and the process, and the process is basically… I would suggest… process is the way forward to the outcome.

I want you to have every confidence that I’m going to treat you no different to everyone else. And the reason I’m doing it isn’t simply the law but I follow the principles of law.

Animal management processes are generally scalable, varying in intensity depending on the circumstance and the discretion of the animal management officer or the particular council. In some cases, a process-based approach is undertaken as there is a requirement to comply with state and local regulations and no ‘discretionary’ funding exists to do other activities; that is to say, the lack of resourcing precludes a more proactive approach. In other cases, a process-based approach is undertaken by choice as the role of animal management in regulating animals is understood to be predicated on a foundation of consistency.

A minority of councils in the sample adopted a more education-oriented approach. Nevertheless, animal management officers from these councils recount a greater focus on outcomes rather than process in the doing of their animal management work:

In regards to enforcement, it’s a very fine line in regards to ‘we’re damned if we do, we’re damned if we don’t’… It depends on what the offence is, but we have a process in place that governs how we enforce, but we do like to be fair and reasonable.

[Situations can] become quite political and I would have to think carefully what the politics of the situation were in making that decision. Even if we had a manual, I couldn’t just go to the manual. So it’s very much outcomes/politics rather than procedures and policies.

These accounts infer an association exists between a council’s predominant education methodology and an institutional outcomes ontology. The first interviewee acknowledges the need to enforce however couching that within a need for a ‘fair and reasonable’ outcome. The second interviewee speaks of politics as driving that council’s focus on outcomes rather than a strict application of process. There is however little evidence suggesting why councils may choose to focus on outcomes aside from the existence of a choice to do so. This contrasts with a real or perceived lack of choice for councils which choose to focus predominantly on enforcement and process. The relative power of councils to choose their approach to animal management appears to drive the ontological and methodological functions in practice. At a cursory glance, this observation may appear to be nuance; however if true, then the choices councils have has a direct influence on the effectiveness of their animal management functions.

### The effectiveness of animal management in practice

To explore this animal management framework in practice, institutional discourse from interviewee accounts is examined with a view to understanding why particular regulatory approaches are more or less effective in particular circumstances. The degree and nature of regulation of dogs importantly affects their welfare in the community. In reflecting and reflexively driving community expectations, regulation affects how dogs are perceived by the broader community and how that perception translates into freedoms afforded to dogs.

Through the provisions of the Act, councils are empowered to create regulations to manage dogs in the public and private realms of their communities, including off-leash controls, effective control and other ordinance. The freedom granted by the Victorian government to councils to manage dogs and provide amenity and services for dogs leads to a range of outcomes driven by each council’s ontological alignment and how that council chooses to manage and accommodate dogs in their communities. In particular, the Act is silent in terms of how councils ought to provide amenity and services for dogs. This silence is supported by accounts from Victorian government representatives who acknowledge of a lack of government appetite to prescribe any uniformity in amenity:

But [the councils] are all contiguous, so why don’t they just have all the same laws?

So you could move a little bit… You could actually start to require more provision for something to be done, but I don’t think the [Victorian] government would want to be in the position of dictating to the councils what services they should provide to their community. If anything, the state government, well my department, would probably prefer it to be somewhere else because the *Domestic Animals Act* brings in all those restricted breed dog/controversial issues, puppy farms and control.

There is substantial variation in approach to animal management across individual councils. Those councils which adopt an outcomes-based institutional ontology differ in approach to animal management however this difference appears largely borne from the situational context and local political ramifications. On the other hand, those councils which adopt a process-based institutional ontology comparatively vary in approach however the reason for such variation in process-based approaches appears arbitrary.

In contrast to institutional ontology, the methodological function of animal management delineates different kinds of regulation and activity. The nature of those activities and that regulation is relatively consistent across councils in practice, this consistency appears largely borne from the smoothing influence of the Act on the making of local regulations to manage dogs. This introduces an apparent orthogonality of ontological and methodological functions of council regulation, however the following examination of council regulation of dogs shows that the effectiveness of the methodological function is related to the underlying ontological function of council regulation in practice.

Accordingly, the remainder of this paper examines four representative regulatory activities of animal management, each of these activities classified by the author according to its predominant ontological and methodological inclinations (see Table 2). These activities are presented as short vignettes of process- and outcomes-based animal management which have been inferred directly from the institutional discourse. This analysis demonstrates how institutional ontology and methodology influence the practice of animal management, providing animal management officers and councils a means to critically examine how particular approaches to animal management work, thereby offering an opportunity to improve the effectiveness of animal management functions in council.

**Table 2:** Common activities of council classified by their ontological (process-outcomes) and methodological (enforcement-education) inclinations (Source: author)

	Process	Outcomes
Enforcement	Barking dog nuisance regulation	Effective control nuisance regulation
Education	Community education programs	Responsible dog ownership

### Process-based animal management

Barking dog nuisance is typically enforced in a process-oriented manner and has some characteristics which distinguish it from other nuisance, most importantly the strong presence of hearsay evidence since the nuisance commonly takes place when the owners are not present. This additional burden of proof can cause friction between neighbours and the problem can persist for some time before official processes begin. An animal management officer illustrates the burden of proof on the complainant by stating that ‘the obligation is to raise the issue with council prior to it becoming a big issue’, begging the questions of what barking dog nuisance is and how the community is able to know when to raise such an issue with council.

The definition of barking dog ‘nuisance’ is not specified in the Act and in practice lacks consensus in both council and the community. Commonly the definition of barking dog nuisance is determined by animal management officers who typically position barking infractions on a subjective scale of frequency and intensity of bark, the calibration of this scale being at the sole discretion of the council or, perhaps, the individual officer. On the other hand, some councils do away with the definition of nuisance almost entirely, adopting a naturalistic stance which accepts that each nuisance is unique and no one definition or scale can capture as such; as one animal management officer expressed, barking dog nuisance is entirely subjective: ‘It’s not how often it [the dog] barks, it’s not when it barks: it’s what that bark does to affect your life’. The definition of nuisance and the nature of the burden of proof are intricately related.

In cases where the council and animal management officers define the nature of nuisance, the complainant is typically burdened to demonstrate that a particular barking incident meets that definition of nuisance. However if the complainant’s definition of nuisance is accepted *prima facie*, then the burden of proof transfers to the respondent to demonstrate that the complaint is manifestly unreasonable. In other words, regardless of how barking dog ‘nuisance’ is defined and consequently where the burden of proof lies, the enforcement process of council remains able to proceed accordingly. An interesting corollary then emerges which posits that no requirement for a clear or consistent definition of barking dog nuisance is necessary for the successful enforcement of barking dog nuisance because of the consistency in application of an enforcement process, whatever that process happens to be. Positive interventions which flow from the successful application of this enforcement process then reduce the incidence or severity of the nuisance, resulting in a more harmonious neighbourhood and a successful enforcement outcome.

The enforcement process clearly plays a central role in the successful treatment of barking dog nuisance. The application of consistent process does not however seem to work nearly as effectively in the context of an educational treatment. Community education programs are a process-based activity of the Victorian government which communicate an educational message of broad appeal to a large audience, the audience catchment greatly exceeding that of a typical council educational program. Whereas the Victorian government’s communication objectives can be satisfied with a process-based approach to education, it remains to examine whether such community education programs can meet a program’s underlying educational objectives; arguably, the success of community education programs ought to prioritise the educational content rather than the success of the program delivery mechanism.

Community education programs are a passive educational process of the Victorian government that ostensibly fulfil requirements under the Act. The programs are designed in coordination with other educational programs run by individual councils, those programs generally targeting school-age children and their guardians. Community education programs typically focus on safety around dogs with a particular focus on preventing the human incidence of dog bite and attack in the community, together with general advice on responsible dog ownership. Importantly, the programs usually focus on human welfare rather than the prevention of dog-to-dog bites and attacks. Each community education program is generally delineated by species and run as an isolated program over a long timeframe, relying in large part on the execution of the education programs documented in each council’s *Domestic Animal Management Plan*. Due to each *Plan*’s four year duration and the inconsistent commitment to resourcing animal management across councils, these documented council-run education programs may bear little relation to what education programs councils actually implement. Consequently, the Victorian government’s reliance on these programs in the formulation of their community education programs inadvertently exposes the community to gaps in awareness and a heightened risk of dog bite. The Victorian government-run community education programs themselves have long lead-times and, due to their necessarily broad appeal, suffer from a tenuous linkage to any actual downstream community benefits. Unsurprisingly then, the Victorian government-run community education programs are typically subject to fickle political commitment with no clear outcome in mind at commencement.

The lack of calibration to particular community outcomes from the beginning underscores the process-driven nature of Victorian government-run community education programs. Furthermore, support for effective community education ought to be self-reinforcing however given the lack of linkage between community education output and the community outcomes which may result from that educational output, there is limited scope for such efforts to demonstrate measurable results. Lacking a foundation of support, the effectiveness of community education programs is further impeded as sufficient funding becomes difficult to obtain due to the lack of easily attributable benefits. If the education programs were instead focused on achieving particular outcomes rather than executing education as a process, the stronger link ought to create a number of positive self-reinforcing effects, including an increase in community support and an increase in the amount and surety of funding, leading to a more effective program overall.

The comparison of these two animal management activities suggests a relationship exists between the effectiveness of a methodology and institutional ontology. Enforcement of a particular nuisance, in relation to barking dog nuisance, has the process itself as driving an outcome. So long as the process has been followed, the community ought to accept the eventual outcome since they have in effect agreed to the process. In terms of community education on the other hand, a process is a matter of necessity by virtue of the sheer scale of audience which the programs aim to capture. The underlying educational message to the community is diluted by this scale, leading to a situation where the communication of the message appears to succeed because of the diligent execution of the process, but the actual message itself appears ineffective because it is not focused on an easily attributable educational outcome.

### Outcomes-based animal management

In contrast to barking dog nuisance, effective control nuisance does not generally engender a common understanding of process or treatment across different councils. Effective control is a general notion that dogs need to be controlled by their owners when engaged in activity in the public realm. Effective control itself is perhaps most distinctively characterised by a complete lack of agreement on the fundamental nature of effective control, let alone agreement on what effective control ‘nuisance’ might be, making effective control nuisance particularly difficult to enforce. This circumstance is relatively common in practice, with Howell’s (2012) account of enforcement in nineteenth century London for instance finding that ‘what constituted the proper control of a dog was… always at best a moot point’ (at page 234). With this in mind, it is hardly surprising that the Act perversely requires councils to enforce ‘effective control’ without articulating precisely what ‘effective control’ means in practice.

Irrespective of whether a dog bark is a nuisance or not, the notion of ‘dog barking’ is within a commonly understood vernacular. In contrast to barking dog nuisance, effective control nuisance fundamentally lacks definitional consensus at any level. The range of interpretations of ‘effective control’ and ‘effective control nuisance’ leads to a range of outcomes for dogs, each affording dogs different opportunities to realise their potential and, consequently, contributing to confusion between council and community as to what effective control is and how it ought to be enforced as the following accounts from animal management officers illustrate:

One of the challenges in a compliance area with dog issues is managing people’s expectations about what effective control is.

Effective control is one of those contentious issues where you might book somebody for having their dog off-lead in an off-leash area and they’ll still struggle to understand why they’ve received an infringement notice.

Since councils and the community may have different expectations over precisely what ‘effective control’ is, it remains unsurprising that effective control lacks successful prosecution in practice begging the question of whether this is driven by a lack of explicit definition or whether a lack of successful enforcement in practice negates any need to develop a definition in the first place.

If it is the lack of explicit definition which makes effective control so difficult to enforce, then simply prescribing a closed-form definition of ‘effective control’ and ‘effective control nuisance’ ought to make enforcement much simpler. This in turn should better align community expectations with those derived from the Act and reduce any conflict arising from misalignment of expectations. The vague construction of effective control at a conceptual level however undermines any efforts to prescribe such a definition. Relaxing any requirement to define effective control ought to ease this tension. Alternatively, if effective control is acknowledged as a normative statement, then the need to prescribe a definition disappears since the role of (strict and precise) enforcement becomes one of (broad and conceptual) education. These typical accounts from animal management officers demonstrate the inherent wickedness of defining effective control:

But this is a debate that I have with people quite a bit in council, not so much with councillors but council officers including the CEO, about why do we need a fully fenced park because they [dogs] are supposed to be under control

The reality of that is that dogs are still dogs and something can distract them. Even the best trained dog can get distracted; it might get chased by another dog. A whole bunch of things can occur that upset the dog.

The first account highlights that there are some council officers who believe that the existence of effective control regulations ostensibly fulfil the purpose of fencing (despite not articulating what effective control means). In contrast, the second account recognises that dogs lack the capability at times to be under the effective control of humans, irrespective of what effective control may mean and, in turn, undermines the imposed requirement to enforce effective control. These two perspectives are irreconcilable and collectively demonstrate that effective control is something which cannot be defined in a closed-form way, making it inherently unenforceable.

Effective control is a desirable attribute of dogs within the community (however it may be defined). In practice, any enforcement of effective control is secondary to any downstream benefits of having effective control (whatever those benefits might be). In other words, effective control is essentially an educational device, differing in structure and ontology from a typical compliance-based object and focused on outcomes or benefits rather than any particular defined process. This infers that the practice of enforcing effective control is failing and in turn suggests an alternative recognition of effective control as an educative device may yield a more effective treatment in practice.

Responsible dog ownership as a notion shares many attributes with effective control, yielding comparable outcomes in society. Both responsible dog ownership and effective control are essentially preventative: if either of these conditions is in place, downstream transgressions and incidents are arguably less severe or may not take place altogether. Neither responsible dog ownership nor effective control prescribe any particular process to generate such outcomes, rather they posit an ideal behaviour. Despite yielding similar outcomes in practice, the treatment of responsible dog ownership and effective control is remarkably different under the Act however. Comparing these treatments consequently provides a window to observe the interplay between institutional ontology and methodology in animal management.

Despite its lack of definition, effective control is treated as a legislative prescription and the Act requires that councils enforce and prosecute infractions. In doing so, the enforcement activity ought to act as both a general and specific deterrent on future transgression and in turn encourage compliance with the effective control requirements (whatever those may be). In contrast, responsible dog ownership is typically treated as an educational device, with both councils and the Victorian government encouraging certain behaviours through education and advice in order to achieve an outcome conducive to a responsible dog owning community:

A lot of compliance-enforcement is about education… so good compliance-enforcement is about trying to tell people because a lot of people actually don’t know. We assume everyone just knows what we know but a lot of people out there go and buy a family pet and often they don’t know a lot of things.

That responsible dog ownership is only loosely defined ‘in principle’ with manifold behavioural composites is unimportant given its educational nature. The lack of definition which afflicts effective control ought to similarly lose relevance if effective control were treated in the spirit of an educational goal rather than as an object for direct enforcement. Such educational treatment would remove the obligation on councils to enforce effective control and consequently remove any need to precisely articulate what effective control is, in turn better aligning community expectations of council with what council actually do. Such alignment of expectations should remediate any detrimental impact on the community perception of council which this failure to enforce effective control may have had. In turn, this ought to improve the community perception of dogs and dog owners, improving their stake in the community.

## Conclusion

This paper introduced a framework to understand the animal management function of councils through the two dimensions of methodology and institutional ontology. Animal management has evolved over the years from the stereotypical role of ‘dog catcher’ with a range of objectives that regulate both the control and inclusion of dogs in society. These objectives are relatively similar across different councils yet how the regulation of dogs is achieved in practice varies quite significantly. This paper employs this methodology-ontology framework to interpret these approaches to regulation and to understand why certain approaches may prove more effective than others across different councils.

The effectiveness of the methodological function is related to the underlying ontological function of council regulation. This result has important practical implications for policymakers and legislators that wish to regulate dogs in urban areas. Not every interaction between dogs and the community can be regulated, nor can every interaction be overlooked. If the approach to a particular infraction is to enforce rather than educate, then this paper suggests that the council adopt a process-oriented approach to undertake that function. On the other hand, if the approach is to educate rather than enforce, then this paper suggests that the council adopt an outcomes-oriented approach to undertake that function. In this paper, the successful examples of barking dog enforcement and responsible dog ownership education illustrate the traits of each approach and why they work. The contrasting unsuccessful examples of effective control enforcement and community education programming illustrate some attributes of failure.

This paper asserts that those councils which adopt a more process-oriented approach ought to have greater success with enforcement activity. In contrast, those councils which adopt a more outcomes-oriented approach ought to have greater success with educational activity. In either case, the expectations of the community are more easily met as the council is acting in accordance with its stated objectives when performing its animal management function; this in turn arguably improves the perception of dogs and their owners in an environment of greater harmony between community and council. Issues arise and conflict emerges when councils lose that discretion and are forced to perform their animal management in a manner which is unaligned with their underlying institutional ontology.

As with other functions of council, the community have certain expectations of the role which animal management ought to fulfil. The community expect the council to regulate compliance with requirements of the Act and may also have other more general expectations which broadly reflect societal norms of how dogs ought to feature. If the council is seen to not fulfil its functions, there is a risk that the broader community’s expectations are not met which exposes both the council and dogs and their owners to potential conflict. This paper provides a number of reflexive insights for councils of where community expectations are not being met and suggests ways in which this could be addressed. Improving the awareness of councils of the importance of how they plan for and manage other species can only be of benefit.

## Footnotes

1 Moreland chose not to furnish the author with their *Domestic Animal Management Plan* and so has been excluded from the study. All other 30 metropolitan councils supplied a copy of their *Domestic Animal Management Plan*.

2 Wyndham chose not to participate in animal management interviews. Nevertheless, their institutional documents were included in the analysis and they remained in the sample of councils.
